# Surgical Repair of an Atraumatic Avulsion of Patellar Tendon at the Tibial Tuberosity in an Adult Patient

**DOI:** 10.1155/2015/192023

**Published:** 2015-06-22

**Authors:** Lorenzo Maria Di Giacomo, M. Shahid Khan, Michele Bisaccia, R. Rende, G. Rinonapoli, A. Caraffa

**Affiliations:** ^1^Division of Orthopedics and Trauma Surgery, University of Perugia, S. Maria della Misericordia Hospital, Via Lorenzini 16, 06123 Perugia, Italy; ^2^Shah Bhitai District Government Hospital, Hyderabad 71000, Pakistan; ^3^University of Perugia, S. Maria della Misericordia Hospital, Via Massari 5, 06122 Perugia, Italy

## Abstract

Atraumatic avulsion of the tibial attachment of patellar tendon in adults is a very rare injury with only few published case reports. Here we are sharing the successful management and follow-up of a similar case with a different suture material for repair of the tendon, the FiberWire. We believe that the management we are discussing allows for early return to activity with good functional outcome.

## 1. Introduction

Isolated avulsion of patellar tendon from tibial tuberosity is very rare injury in the adults [1]. Spontaneous patellar tendon rupture may result from steroid use, systemic metabolic and inflammatory conditions [2] but these injuries mostly occur at the inferior pole of the patella [2]. As per literature isolated spontaneous complete distal avulsion of the patellar tendon in an otherwise healthy patient is very rare so here we are sharing similar case management with 5-month follow-up. We believe that the management we are discussing allows for early return to activity with good functional outcome.

## 2. Case Presentation

**Figure 1:**
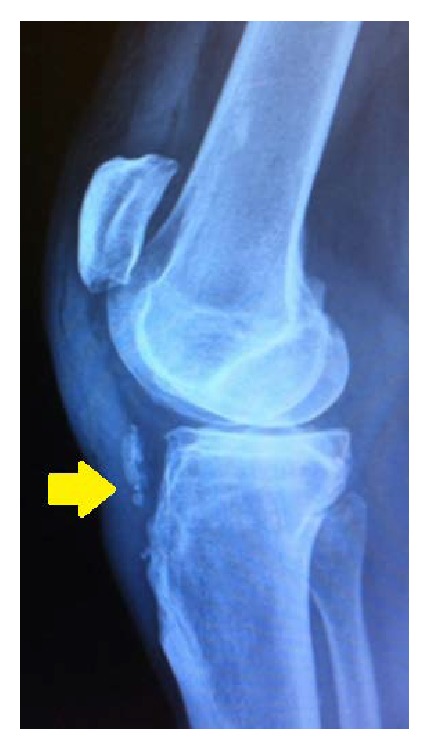
Injury film showing high patella and presence of calcification in the distal part of the patellar tendon (arrow).

**Figure 2:**
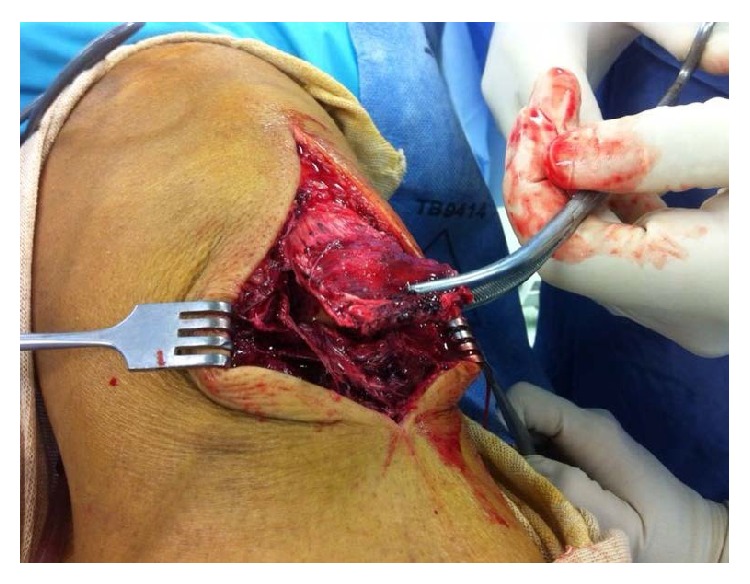
Intraoperative clinical picture showing avulsed patellar tendon from tibial tuberosity.

**Figure 3:**
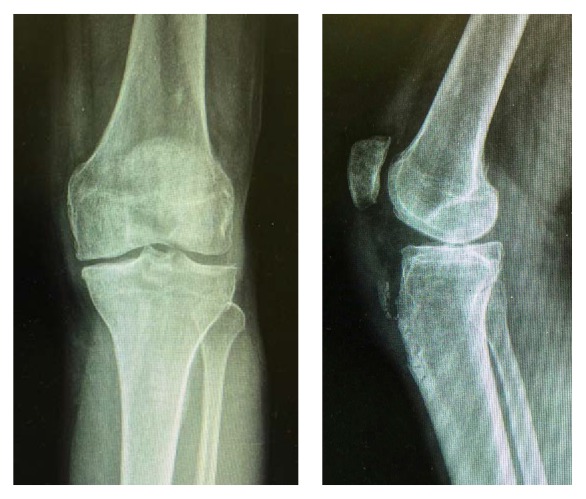
Knee X-ray at 5-month follow-up.

52-year-old man, plumber by profession, presented at the emergency department with one day history of having sudden onset spontaneous pain and functional impairment of left knee while he was walking. The patient had history of hypertension. He had no history of taking medications such as corticosteroids or fluoroquinolones and did not remember having suffered any knee pain previously. On clinical examination effusion was present in knee. It was possible to palpate a gap between the distal patellar tendon and the tibial tuberosity and patient was not able to extend the leg. X-ray of the left knee showed a high patella and presence of calcification in the distal part of the patellar tendon (Figure 1). Ultrasound later confirmed the diagnosis of distal avulsion of the patellar tendon. Although CT scan and MRI are superior modalities for confirmation of the diagnosis but based on our clinical examination, typical radiographic finding and ultrasound imaging, we considered these expensive investigations unnecessary in this case. On the second day, patient was taken to the operating room. Under tourniquet control, patellar tendon was exposed through anterior approach to the knee. Distal insertion of the tendon was found to be completely avulsed from the tibial tuberosity with some flakes of sclerotic bone (Figure 2). The torn tendon was found to have degenerative changes at the injury site. The tendon stump was debrided and refashioned. Locking double Krackow stitch was taken with FiberWire number 5, and then it was anchored to tibial tuberosity after drilling four 2.0 mm transverse holes in tuberosity. Full range of motion of the knee was performed to evaluate the repair and we found it satisfactory. Repair of paratenon was performed and wound was closed. Postoperatively, knee was locked in a brace at 0 degrees of extension and the patient was encouraged to do isometric quadriceps strengthening exercises. Patient was allowed weight bearing as tolerated. A range of motion exercises were progressively started after 5-week follow-up. The patient achieved full active range of motion by third month of follow-up, complaining only of a slight tenderness on pressure at the level of the tibial tuberosity. X-rays were taken in the last follow-up at 5 months showing normal position of the patella (Figure 3), while clinical examination revealed full recovery.

## 3. Discussion

Patellar tendon rupture occurs almost exclusively in males as a consequence of landing after a fall or a jump which produces a rapid contraction of the quadriceps muscle with a partially flexed knee or a consequence of direct trauma to the knee [2]. Mostly the rupture occurs at the inferior pole of the patella; however, distal avulsion is very uncommon [2]. Multiple conditions may predispose the tendon rupture with relatively minor injury. These comorbid conditions include diabetes, rheumatoid arthritis, corticosteroid therapy (either systemic or local injection), chronic kidney insufficiency, fluoroquinolone treatment, and hyperparathyroidism.

Management of patellar tendon rupture varies from primary repair strengthened by cerclage augmentation and immobilization of the extension for 6 weeks [3, 4] to primary repair without augmentation and allowing early, passive controlled range of motion from the beginning without any immobilization [5]. Good results have been documented with the former technique but it may lead to limited flexion, weakness, patella infera, and a second surgery for hardware removal [3, 5]. Latter technique claims faster, less restricting rehabilitation and a shorter time for return to activity [5] but this technique demands highly compliant patients and highly specialized rehabilitation staff as well. Keeping in mind the pros and cons of both abovementioned approaches we opted to use FiberWire for the repair of the tendon followed by immobilization of the knee in a brace for 5 weeks. We trusted the strength of the FiberWire as it has been shown that a single loop of number 5 FiberWire has the closest material properties to the 18-gauge stainless steel wire. In addition, FiberWire does not require future removal and it has the advantage of being less irritating to the surrounding tissues as compared to the stainless steel wire [6]. We found only one case report of complete avulsion of the patellar tendon of the tibial tubercle in adults in the literature by Chloros et al. [1]. Like their study we too are unable to have an explanation of spontaneous rupture patellar tendon as the patient had no previous complaint of pain and neither had he any predisposing comorbid conditions. Chloros et al. used Ticron sutures and G2 Mitek anchor for the repair of tendon and kept limb in a hinged brace locked in full extension postoperatively for 6 weeks with weight bearing as tolerated. They started progressive range of motion exercises from 6 weeks and showed good results with a follow-up of two and a half months. Suture material we opted for the repair is more biocompatible [6] and in combination with starting relatively early range of motion exercises we were able to achieve good functional outcome with a follow-up of 5 months.

Potential complications with use of FiberWire are rupture of the sutures, increased tendinosis by the sutures, and reduction of the strength in knee extension. One of the rare potential complications described in literature with use of FiberWire is reaction to the synthetic material leading to discharging sinuses. In our case, none of the abovementioned complications was observed.

## 4. Conclusion

Avulsion of distal insertion of the patellar tendon is an extremely rare injury in adults that requires reliable fixation followed by supervised rehabilitation to get the best possible functional outcome. There are very few case reports in the literature describing surgical repair of these types of injuries. In contrast to conventional treatment options, our management of reinsertion of the tendon using transosseous suture with FiberWire is an attractive alternative treatment option that provides excellent resistance combined with good biocompatibility.
